# Second allogeneic hematopoietic stem cell transplantation in patients with inborn errors of immunity

**DOI:** 10.1038/s41409-022-01883-4

**Published:** 2022-12-01

**Authors:** Alexandra Laberko, Elvira Sultanova, Aishat Idarmacheva, Yulia Skvortsova, Larisa Shelikhova, Alexei Nechesnyuk, Daria Kobyzeva, Anna Shcherbina, Michael Maschan, Alexei Maschan, Dmitry Balashov

**Affiliations:** 1 Department of Immunology, Dmitry Rogachev National Medical Research Center of Pediatric Hematology, Oncology and Immunology, Moscow, Russia; 2 Translational and Clinical Research Institute, Newcastle University, Newcastle-upon-Tyne, UK; 3 Department of Hematopoietic Stem Cell Transplantation, Dmitry Rogachev National Medical Research Center of Pediatric Hematology, Oncology and Immunology, Moscow, Russia; 4 Department of Radiation Therapy, Dmitry Rogachev National Medical Research Center of Pediatric Hematology, Oncology and Immunology, Moscow, Russia

**Keywords:** Stem-cell research, Stem-cell therapies

## Abstract

Graft failure (GF) remains a serious issue of hematopoietic stem cell transplantation (HSCT) in inborn errors of immunity (IEI). Second HSCT is the only salvage therapy for GF. There are no uniform strategies for the second HSCTs and limited data are available on the second HSCT outcomes. 48 patients with various IEI received second allogeneic HSCT from 2013 to 2020. Different conditioning regimens were used, divided into two main groups: containing myeloablative doses of busulfan/treosulfan (*n* = 19) and lymphoid irradiation 2–6 Gy (*n* = 22). Irradiation-containing conditioning was predominantly used in suspected immune-mediated rejection of the first graft. Matched unrelated donor was used in 28 patients, mismatched related in 18, and matched related in 1. 35 patients received TCRαβ/CD19 graft depletion. The median follow-up time was 2.4 years post-HSCT. One patient died at conditioning. The OS was 0.63 (95% CI: 0.41–0.85) after busulfan/treosulfan and 0.68 (95% CI: 0.48–0.88) after irradiation-based conditioning, *p* = 0.66. Active infection at HSCT significantly influenced OS: 0.43 (95% CI: 0.17–0.69) versus 0.73 (95% CI: 0.58–0.88) without infection, *p* = 0.004. The cumulative incidence of GF was 0.15 (95% CI: 0.08–0.29). To conclude, an individualized approach is required for the second HSCT in IEI. Low-dose lymphoid irradiation in suspected immune-mediated GF may be a feasible option.

## Introduction

Allogeneic hematopoietic stem cell transplantation (HSCT) is an effective curative modality for many patients with inborn errors of immunity (IEI) (formerly known as primary immunodeficiency). Over the past years, the HSCT outcomes for IEI have been dramatically improving [1]. This is related to many factors, such as a wide use of methods of graft engineering, including TCRαβ/CD19 depletion or post-transplant cyclophosphamide (Pt-Cy) in HLA-mismatched donors, or reduction of conditioning-associated toxicity by administration of treosulfan or busulfan dose adjustment [2].

However, despite reduced HSCT-related toxicity, a serious issue remains graft failure (GF), which can be mediated by recipient immune cells or loss of donor hematopoiesis, owing to other causes that are often unknown. The risk of GF is known to be higher in non-malignant disorders [3]. The incidence of GF in multicenter studies for distinct IEI is reported to vary from 8 to 16%, and up to half of the patients developing GF decease before re-transplantation or after the second HSCT [4–8]. Factors contributing to GF development might be related not only to underlying disease, but also to conditioning regimen, donor/recipient HLA match, graft composition, T-cell depletion, and others [3].

The only therapeutic option for GF is a second allogeneic HSCT; however, historically it has been associated with high treatment-related morbidity and mortality. A choice of approach for the second HSCT is rather challenging, there are no uniform strategies of selection of conditioning regimen or donor. Another challenge is the timing of re-transplantation due to the balance between the risks of cumulative toxicity of two HSCTs. Moreover, post-HSCT prolonged bone marrow (BM) aplasia or secondary immunodeficiency often predispose to life-threating infections, which is of particular importance for IEI patients, often having pre-first HSCT infections and associated organ damage. The published data on the second HSCT in non-malignant diseases are limited by few studies in small groups of patients.

In the current study, we analysed the outcomes of second HSCTs in a group of patients with IEI.

## Methods

Engraftment and GF, which included the cases of primary GF and graft rejection were registered as described previously [9]. From 2012 to 2020, 312 patients with IEI underwent a first HSCT in our center, and 52 (17%) of them developed GF. Forty-eight patients with IEI received a second HSCT procedure in our center between 2013 and 2020. The first HSCT details are shown in Table S1 supplement, main patients’ characteristics and second HSCT details are shown in Table 1.

**Table 1:** Characteristics of patients and HSCTs.

Patient №	Molecular diagnosis	Age at 2nd HSCT, years	Time from 1st to 2nd HSCT, months	Indication for 2nd HSCT	Morbidity at HSCT	Conditioning regimen			Donor (HLA-match)	Graft type	Post-HSCT immune-suppression	Follow up, time
						group	type	drugs and doses				
1	CD40L	3.0	8.0	EGF	–	TAI/TLI	RIC	TAI6, Flu150, Cy120	MUD (10/10)*	PBSC, TCRαβ depletion	Tacro, Mtx	Alive, 9 years
2	CYBB	6.3	8.4	EGF	–	Bu/Treo	MAC	Bu16, Flu150, Alemt2	MUD (10/10)	BM	Tacro, MMF	Alive, 8.7 years
3	XIAP	5.3	6.5	EGF	CMV viremia, pneumonia undefined, active HLH	Bu/Treo	MAC	Treo36, Flu150, Thymo5, VP60, Rit	MUD (10/10)	PBSC, TCRαβ depletion	Steroids, Mtx	Death day +52 (Ps. aeruginosa sepsis)
4	ELANE	9.3	3.5	EGF	CMV viremia, agranulocytosis	Bu/Treo	MAC	Bu12, Flu150, Cy120, Alemt1, Rit	MUD (10/10)	BM	Tacro, Mtx	GF day +22, death day +36
5	CID undefined	4.6	4.8	EGF	massive hepatomegaly	Bu/Treo	MAC	Bu12, Flu150, Cy120, Alemt1	MMRD (6/10)	PBSC, TCRαβ depletion	Tacro, Mtx	Alive, 8.1 years
6	STAT1 LOF	6.5	2.1	EGF	BM aplasia	lymphoid irradiation	MAC	TLI6, Flu150, Thymo5, Cy120, Mel140, Rit	MMRD (5/10)	PBSC, TCRαβ depletion	Tacro, Mtx	Alive, 7.9 years
7	WAS	2.3	3.6	EGF	severe thrombocytopenia	lymphoid irradiation	RIC	TLI6, Flu150, Thymo5, Thio10	MUD (10/10)*	PBSC, Pt-Cy	Tacro	GF day +60, 3^rd^ HSCT, alive 7.8 years
8	IL2RG	1.7	13.3	LGF	–	Bu/Treo	MAC	Treo36, Flu150, Alemt1, Cy100, Rit	MUD (10/10)	PBSC, TCRαβ depletion	Tacro	Alive, 7.8 years
9	ELANE	1.4	3.5	EGF	agranulocytosis	lymphoid irradiation	MAC	TLI4, Flu150, Alemt0.5, Thio10, Mel80	MMRD (6/10)	BM, Pt-Cy	Tacro	GF day +66, death 1.4 years
10	RMRP	3.3	3.5	EGF	–	Bu/Treo	MAC	Treo42, Flu150, Alemt1, Thio10	MUD (10/10)	PBSC, TCRαβ depletion	Tacro, Mtx	Alive, 7.3 years
11	HLH undefined	2.8	4.7	EGF	CMV viremia	–	–	Treo42, Flu150, Cy30	MUD (10/10)	BM, Pt-Cy	Tacro	Alive, 7.2 years
12	WAS	1.2	3.4	EGF	severe thrombocytopenia	lymphoid irradiation	RIC	TAI4, Flu150, Thymo5, Cy30, Mel140	MUD (9/10)*	PBSC, Pt-Cy	Tacro	GF day +244, 3^rd^ HSCT, alive 7 years
13	CYBB	15.1	3.0	EGF	–	lymphoid irradiation	MAC	TAI6, Flu150, Thymo5, Cy120, Thio10, Rit	MUD (9/10)*	PBSC, TCRαβ depletion	Tacro	Alive, 6.8 years
14	UNC13D	1.5	4.1	EGF	–	lymphoid irradiation	MAC	TAI4, Flu150, Thymo2.5, Cy120, Mel140, Rit	MUD (10/10)*	PBSC, TCRαβ depletion	Tacro	Alive, 6.7 years
15	IL2RG	4.0	15.2	absent immune recovery	liver fibrosis due to cGVHD	lymphoid irradiation	RIC	TAI4, Flu150, Thymo5, Treo36, Rit	MUD (10/10)	PBSC, TCRαβ depletion	Steroids	Death day +406 (Candida sepsis)
16	RAG1	1.7	16.5	LGF	–	Bu/Treo	MAC	Treo42, Flu150, Thymo5, Mel140, Rit	MUD (10/10)	PBSC, TCRαβ depletion	Abat, Mtx	Death day +201 (HHV6 infection)
17	CID undefined	16.8	7.7	EGF	–	lymphoid irradiation	RIC	TAI2, Flu150, Thymo5, Cy30	MMRD (5/10)	BM, Pt-Cy	Tacro	Death day +115 (sepsis undefined)
18	WAS	1.8	8.8	LGF	severe thrombocytopenia	Bu/Treo	MAC	Treo42, Flu150, Thymo5, Thio10, Plerix/G-CSF, Rit	MUD (9/10)	PBSC, TCRαβ depletion	Tacro	Alive, 5.8 years
19	WAS	3.8	7.9	EGF	severe thrombocytopenia	Bu/Treo	MAC	Treo42, Flu150, Thymo5, Thio10, Plerix/G-CSF, Rit	MUD (10/10)	BM	Tacro, MMF	Death day +533 (GVHD)
20	WAS	2.1	7.3	LGF	severe thrombocytopenia	Bu/Treo	MAC	Treo42, Flu150, Thymo5, Mel140, Plerix/G-CSF, Rit	MUD (10/10)	PBSC, TCRαβ depletion	Tacro	Alive, 5.4 years
21	IL2RG	2.2	15.1	absent immune recovery	Norovirus, rotovirus colitis, lung cGVHD?	Bu/Treo	MAC	Treo36, Flu150, Thymo5, Mel140, Rit	MMRD (6/10)	PBSC, TCRαβ depletion	Abat, Toc	Death day +11 (sepsis undefined)
22	IL2RG	1.4	4.6	EGF	–	Bu/Treo	MAC	Treo36, Flu150, Thymo5, Thio10	MUD (9/10)	PBSC, TCRαβ depletion	Abat, Toc	Alive, 5.3 years
23	WAS	3.1	13.0	LGF	severe thrombocytopenia	Bu/Treo	MAC	Treo42, Flu150, Thymo5, Thio10, Plerix/G-CSF, Rit	MUD (10/10)	PBSC, TCRαβ depletion	Tacro	Alive, 4.9 years
24	NBN	5.8	11.3	LGF	–	–	–	Treo30, Flu150, Thymo5, Cy40, Rit	MUD (10/10)	PBSC, TCRαβ depletion	Tacro	Alive, 4.8 years
25	NBN	10.6	9.1	EGF	–	–	–	Treo30, Flu150, Thymo5, Cy30, Rit	MUD (10/10)	PBSC, TCRαβ depletion	Tacro, Mtx	Alive, 4.8 years
26	IL2RG	0.9	4.8	EGF	disseminated CMV, RS pneumonia	Bu/Treo	MAC	Treo42, Flu150, Thymo5, Thio10, Rit	MUD (10/10)	PBSC, TCRαβ depletion	Abat, Toc	Death day +9 (Ps. aeruginosa sepsis)
27	CYBB	4.2	17.3	LGF	–	Bu/Treo	MAC	Treo42, Flu150, Thymo5, Thio10, Plerix/G-CSF, Rit	MUD (10/10)	PBSC, TCRαβ depletion	no	Alive, 3.8 years
28	ELANE	5.4	3.2	EGF	agranulocytosis	Bu/Treo	MAC	Bu16, Flu150, Thymo5, Thio10, Plerix/G-CSF, Rit	MRD (9/10)	PBSC, TCRαβ depletion	CsA	Alive, 3.6 years
29	CTLA4	17.0	3.8	EGF	–	lymphoid irradiation	MAC	TAI4, Flu150, Thymo10, Cy120, Mel140, Rit	MMRD (5/10)	PBSC, TCRαβ depletion	no	GF day +61, death day +103
30	LIG4	9.3	7.9	EGF	therapy-related endothelial toxicity, BM aplasia	–	–	Flu150, Thymo5, Cy40, Rit	MMRD (6/10)	PBSC, TCRαβ depletion	Abat, Toc	Death,day +107 (endothelial cell damage)
31	XIAP	2.9	4.2	EGF	inflammatory bowel disease	lymphoid irradiation	MAC	TAI3, Flu150, Thymo10, Cy120, Mel180, Rit	MMRD (5/10)	PBSC, TCRαβ depletion	CsA	Alive, 3.3 years
32	KRAS	3.9	5.4	EGF	BM aplasia, inflammatory bowel disease	lymphoid irradiation	MAC	TAI4, Flu150, Thymo10, Cy100, Mel180, Rit	MMRD (5/10)	PBSC, TCRαβ depletion	CsA	Alive, 3.2 years
33	CXCR4	1.8	5.1	EGF	–	lymphoid irradiation	MAC	TAI4, Flu150, Thymo5, Cy100, Mel180, Rit	MUD (9/10)	PBSC, TCRαβ depletion	CsA	GF day +77, death day +154
34	ATM	1.3	3.2	EGF	CMV viremia, BM aplasia	–	–	Flu150, Thymo5, Thio10, Plerix/G-CSF, Rit	MMRD (5/10)	PBSC, TCRαβ depletion, Pt-Cy	no	Death day +61 (pneumonia undefined)
35	HLH undefined	1.2	1.6	EGF	necrotizing stomatitis, BM aplasia	–	–	Flu100, Thymo10, Cy100	MMRD (7/10)*	PBSC, TCRαβ depletion	no	GF day +91, alive 2.7 years
36	IL2RG	2.7	8.5	LGF	-	Bu/Treo	MAC	Treo42, Flu150, Thymo5, Thio10, Rit	MUD (9/10)	PBSC, TCRαβ depletion	Abat, Toc	Alive, 2.6 years
37	SCN undefined	3.5	6.6	BM aplasia	CMV viremia, BM aplasia	lymphoid irradiation	MAC	TAI6, Thymo5, Cy120, Thio10, Rit	MUD (10/10)	PBSC, TCRαβ depletion	Abat	Alive, 2.4 years
38	ELANE	2.1	3.6	EGF	agranulocytosis	lymphoid irradiation	MAC	TAI6, Flu150, Thymo5, Cy120, Mel180, Rit	MMRD (5/10)	PBSC, TCRαβ depletion	no	Alive, 2.4 years
39	CD40L	3.3	23.8	EGF	–	lymphoid irradiation	MAC	TAI6, Flu150, Thymo5, Cy120, Mel180, Rit	MMRD (5/10)	PBSC, TCRαβ depletion, Pt-Cy	CsA	Alive, 2.3 years
40	PSTPIP1	9.6	5.5	EGF	CMV viremia, BM aplasia	lymphoid irradiation	MAC	TLI6, Flu150, Thymo5, Cy120, Thio10, Rit	MUD (10/10)	PBSC, TCRαβ depletion	no	Alive, 2.1 years
41	KRAS	7.9	2.6	EGF	BM aplasia	lymphoid irradiation	MAC	TLI6, Flu150, Thymo5, Cy120, Mel180, Rit	MMRD (5/10)	PBSC, TCRαβ depletion	no	Alive, 2 years
42	SBDS	17.6	8.1	AML relapse	AML relapse	–	–	ТBI12(TMI15), VP60, Flu150, Rit	MMRD (5/10)	PBSC, TCRαβ depletion	Abat, Toc, Bort	Death day +123 (AML relapse)
43	HLH undefined	1.2	2.0	EGF	CMV, ADV viremia, active HLH	Bu/Treo	MAC	Treo42, Flu150, Thymo5, VP60, Rit	MMRD (5/10)	PBSC, TCRαβ depletion	CsA, Abat, Toc, Rux, Steroids	Death day +38 (reactivation of HLH)
44	WAS	5.4	10.0	LGF	CMV viremia, ADV colitis, skin cGVHD, severe thrombocytopenia	lymphoid irradiation	RIC	TLI6, Flu150, Thymo5, Cy100, Plerix/G-CSF, Rit	MUD (10/10)	PBSC, TCRαβ depletion	no	Alive, 1.6 years
45	GATA2	17.2	4.4	EGF	BM aplasia	lymphoid irradiation	RIC	TLI6, Flu150, ATGAM100, Cy20, Treo36, Rit	MUD (10/10)	BM, Pt-Cy	CsA	Alive, 1.3 years
46	WAS	4.9	45.7	uncontrolled AIHA	Norovirus colitis, AIHA	Bu/Treo	MAC	Bu16, Flu150, ATGAM100, Thio10, Plerix/G-CSF, Rit	MMRD (6/10)	PBSC, TCRαβ depletion	no	Alive, 1.3 years
47	GATA2	15.9	2.6	EGF	Invasive aspergillosis, BM aplasia	lymphoid irradiation	RIC	TLI4, Flu150, ATGAM100, Cy70, Treo36, Rit	MMRD (5/10)	PBSC, TCRαβ depletion, Pt-Cy	Abat	Death day +32 (Invasive aspergillosis)
48	SBDS	15.2	2.5	EGF	Pneumonia undefined, BM aplasia	lymphoid irradiation	RIC	TLI4, Flu150, ATGAM70, Cy120	–	–	no	Death day −1 (capillary leak syndrome)

*HLH* hemophagocytic lymphohistiocytosis, *CID* combined immunodeficiency, *SCN* severe congenital neutropenia, *GF* graft failure, *EGF* first HSCT early graft failure (before day + 100 post-HSCT), *LGF* first HSCT late graft failure (after day + 100 post-HSCT), *BM* bone marrow, *AIHA* autoimmune hemolytic anemia, *RIC* reduced intensity conditioning, *MAC* myeloablative conditioning, *TAI* thoracoabdominal irradiation (Gy), *TLI* total lymphoid irradiation (Gy), *TBI* total body irradiation (Gy), *TMI* total marrow irradiation (Gy), *Cy* cyclophosphamide (mg/kg), *Flu* fludarabine (mg/m2), *Treo* treosulfan (g/m2), *Bu* busulfan (mg/kg), *VP* vepesid (mg/kg), *Thymo* thymoglobulin (Genzyme Europe, mg/kg), *Thio* thiotepa (mg/kg), *Mel* melphalan (mg/m2), *Plerix* plerixafor (0.72 mg/kg), *G-CSF* granulocyte colony-stimulating factor (50 mg/kg), *Alemt* Alemtuzumab (mg/kg), *MUD* matched unrelated donor, *MMRD* mismatched related donor, *MRD* matched related donor, *PBSC* peripheral blood stem cell, *Pt-Cy* posttransplant cyclophosphamide (100 mg/kg, day + 3,4 post-HSCT), *Rit* rituximab (100–375 mg/m2), *Abat* abatacept (10 mg/kg days −1, +7, +14, +28), *Tacro* tacrolimus, *СsA* cyclosporin A, *Мtx* methotrexate (10–15 mg/m2 days + 1, +3, +6, +11), *MMF* mycophenolate mofetil 30 mg/kg/day, *Rux* ruxolitinib, *Toc* tocilizumab (8 mg/kg days −1, +7, +14, +28), Steroids - methylprednisolone 0.5–1 mg/kg/day; *Bort* bortezomib (1.4 mg/m2 days −5, −2, +2,+5)

*the same donor as was used in the first HSCT.

Indications for the second HSCT were: first HSCT primary GF in 9 patients or graft rejection in 34, prolonged BM aplasia with high blood transfusion dependence despite full donor chimerism in 1, long-term absence of immune reconstitution despite full donor whole blood or CD3+ chimerism in 2, autoimmune hemolytic anemia (AIHA) uncontrolled with immunosuppressive therapy and splenectomy in 1, and progression of pre-first HSCT myelodysplastic syndrome (MDS) to acute myeloid leukemia (AML) in 1 (Table 1).

After the first HSCT, 34 of 43 patients developed early GF and 9 patients developed late GF (the details are shown in Table 2). The type of first HSCT GF was defined as lymphoid or myeloid by predominant CD3+ or CD15+ recipient chimerism in peripheral blood before GF, respectively. Three patients had full donor chimerism and two patients split chimerism at the time of the second HSCT.

**Table 2:** Details of early and late graft failure after first HSCT.

Characteristic	Early graft failure (EGF)	Late graft failure (LGF)
Definition	before day 100 post-HSCT	after day 100 post-HSCT
Number of patients	30	13
Primary/secondary GF	9/21	all secondary
Type of GF: lymphoid/myeloid/unknown	10/3/17	4/8/1
Median (range) time at graft rejection after HSCT (days)	29 (16–88)	171 (124–428)
Median (range) time between the first and second HSCT (months)	3.7 (1.6–9.1)	10 (7.3–23.8)
2nd HSCT conditioning regimen: irradiation/ Bu-Treo based	16/11	4/9

*GF* graft failure, *HSCT* hematopoietic stem cell transplantation, *Bu* busulfan, *Treo* treosulfan.

Various conditioning regimens were used, which were divided into two groups: lymphoid irradiation-based in 22 patients (total lymphoid irradiation 4–6 Gy in 9, thoracoabdominal irradiation 2–6 Gy in 13), busulfan/treosulfan-based in 19 patients (including plerixafor-containing in 7); and 7 patients received other regimens (Table 1). Most of the patients received serotherapy and rituximab. Irradiation-based conditioning was predominantly used in patients with first HSCT early GF and busulfan/treosulfan-based conditioning predominantly in patients with late GF (Tables 1 and 2). As irradiation-based conditioning regimens contained a different composition and doses of myeloablative agents, this group was additionally divided into irradiation-containing myeloablative or reduced intensity conditioning (MAC and RIC), which were defined based on Shaw et al. [10] and in cases of individual dosing were determined roughly.

One patient died at conditioning and did not receive HSCT. In 37 patients, various regimens of post-HSCT immunosuppressive therapy were implemented and 10 patients did not receive immunosuppression (Table 1). Matched unrelated donor (MUD) was used in 28 patients, mismatched related donor (MMRD) in 18 patients, and matched related donor in 1 patient. In 41 patients, alternative donors were used for the second HSCTs, and 6 received the second HSCTs from the same donor (marked with asterisk in Table 1). Peripheral blood stem cells (PBSC) were used as a graft source in 35 patients receiving TCRαβ/CD19 graft depletion, and 2 patients receiving Pt-Cy; and 3 patients received PBSC with Pt-Cy after TCRαβ/CD19 depletion due to high number of TCRαβ+ cells in graft. Unmanipulated BM was used in 7 patients, 4 of them received Pt-Cy (Table 1).

Thirty-two of 48 patients experienced severe morbidity prior to the second HSCT: 16 – complications of underlying IEI (2 – inflammatory bowel disease, 7 – thrombocytopenia (all had Wiscott-Aldrich syndrome), 4 – agranulocytosis, 2 – active hemophagocytic lymphohistiocytosis (HLH), 1 – massive hepatomegaly), 14 – uncontrolled infections, 11 – BM aplasia with high transfusion dependence, immune complications of the first HSCT (2- chronic graft versus host disease (GVHD), 1 - AIHA), 1 – first transplant-related endothelial toxicity, 1 – active AML (Table 1). Eighteen of 32 patients had only one morbidity and 14 more than one morbidity at the second HSCT, most of them (*n* = 12) had uncontrolled infection in combination with another complication. In those patients without active infection at HSCT, uncontrolled autoimmune complications (both HSCT and prior disease related), therapy-related toxicity, cGVHD, active malignancy and BM aplasia with high transfusion dependence were considered as serious morbidity.

For monitoring of viral infections, quantitative real-time polymerase chain reaction (PCR) was used. In all patients, regular (once every 1–2 weeks) PCR of peripheral blood was assessed for cytomegalovirus (CMV), Epstein Barr virus (EBV) and human herpesvirus VI (HHV-VI), and in 40 patients was also regularly assessed adenovirus (ADV). The other viruses were assessed additionally, when required. More than 100 copies of CMV or ADV DNA per 1 mL of blood or other biological fluids were considered as infection. In the other detected viruses, only infections requiring antiviral therapy were considered significant. For GVHD diagnosis and grading were used the standard definitions [11]. Organ functions were not fully assessed in all the patients at last follow-up (FU), so that the incidence of late complications may be underestimated.

Statistical analysis was performed with the XLSTAT software (Addinsoft) in April 2022, as described before [9]. The patient deceased at conditioning was included in the analysis of survival and excluded from the analysis of complications risks.

## Results

### Study group

Forty-eight (92%) patients underwent a second HSCT procedure between 2013 and 2020. The median age at second HSCT was 3.65 years (range 0.9–17.6). The median time between the first and second HSCT was 5.3 months (range 1.6–45.7) and between graft rejection and the second HSCT 3.3 months (range 0.7–11.3).

### Engraftment and graft failure

**Fig. 1:**
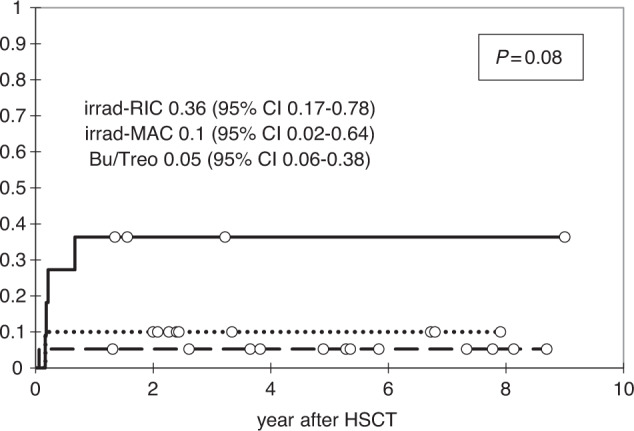
Cumulative incidence of second HSCT graft failure depending on the type of conditioning regimen. myeloablative conditioning (MAC) with irradiation, *n* = 10 (dotted curve) versus reduced intensity conditioning (RIC) with irradiation, *n* = 12 (solid curve) versus busulfan/treosulfan-based, *n* = 19 (dash curve).

From 47 patients, 44 achieved engraftment, 2 patients died before engraftment, and 1 had primary GF. The median time at neutrophil engraftment was 13 days (range 6–32) and platelet engraftment 15 days (range 9–35) after HSCT. Six of 47 patients developed graft rejection, the median time to graft rejection was 2.4 months (range 2–8). The cumulative incidence of GF was 0.15 (95% CI: 0.08–0.29). Four of seven patients with GF died, two of them after the third HSCT. The incidence of GF after irradiation-containing RIC was 0.36 (95% CI: 0.17–0.78), irradiation-containing MAC 0.1 (95% CI: 0.02–0.64), and 0.05 (95% CI: 0.06–0.38) after busulfan/treosulfan-based conditioning, *p* = 0.08 (Fig. 1). In two patients with severe congenital neutropenia, GF was associated with high titres of anti-HLA antibodies due to multiple granulocyte transfusions prior to the first or the second HSCT.

### Acute and chronic GVHD and viral infections

The cumulative incidence of acute GVHD grade ≥II was 0.17 (95% CI: 0.09–0.32). Five patients had grade II, three patients grade III and none grade IV acute GVHD. The cumulative incidence of chronic GVHD was 0.14 (95% CI: 0.07–0.28); three patients had limited and three patients extensive forms.

Thirty six of 47 patients (77%) had significant viral infection after second HSCT. Thirty two (68%) developed CMV infection (9 had active infection at HSCT): CMV viremia in 22 and viremia with organ damage in 10 (retinitis −6, pneumonia −4, colitis −1). Eight patients had ADV infection (one had active infection at HSCT), in all viremia was accompanied by organ involvement (pneumonia −4, colitis −8, urinary system infection −2). Eight patients had other significant infections: HHV6 −4, parvovirus В19 −1, BK virus −1, herpes simplex and herpes zoster −1, post-transplant lymphoproliferative disease (EBV-positive diffuse large B-cell lymphoma) −1.

### Survival and mortality

The median FU time was 2.4 years (range 0–9) after HSCT. In 48 patients, the OS was 0.64 (95% CI: 0.51–0.78). The median time of death was 3.4 months (range 0–17.8) after HSCT. Sixteen patients died of transplant-related mortality, and one patient of AML relapse. The causes of death in 11 patients were infections: bacterial or fungal in 8, viral in 3 (1 – ADV, 1 - HHV6, 1 – CMV). Three patients died of HSCT-related organ toxicity: one with Schwachman-Diamond syndrome (SDS) died at conditioning of chemotherapy-related toxicity, one of chronic GVHD and one of disseminated intravascular coagulation due to virus infection and reactivation of HLH.

**Fig. 2:**
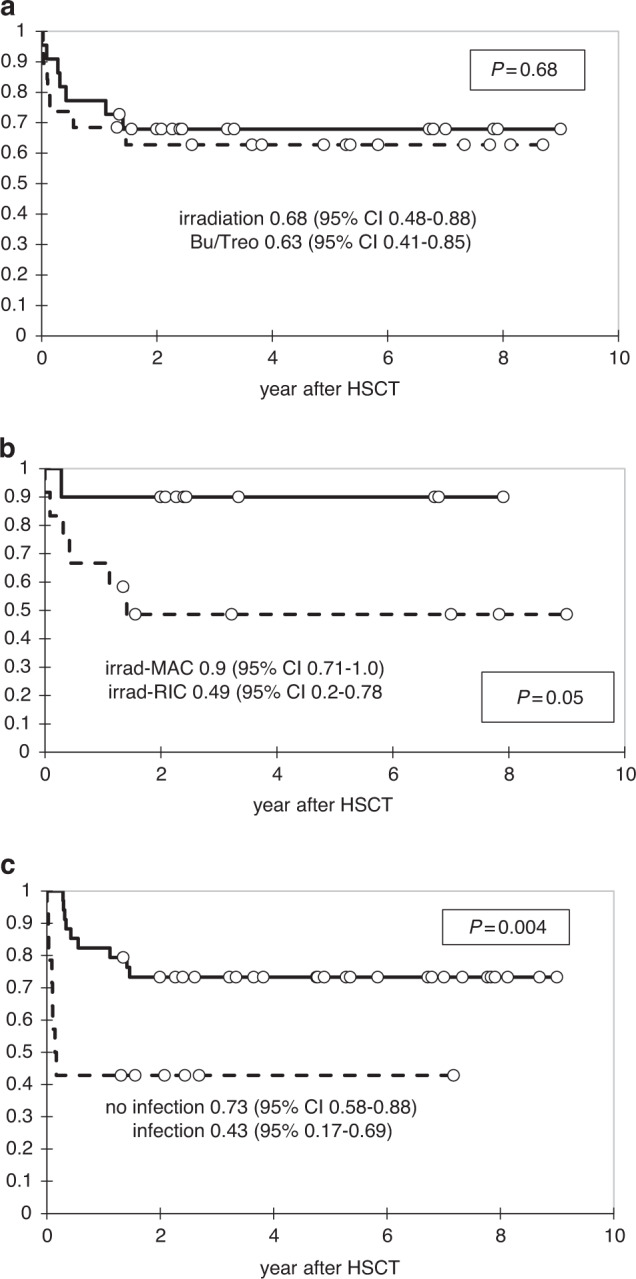
Overall survival of the patients with IEI after second HSCT. **a** Depending on the type of conditioning regimen: irradiation-based, *n* = 22 (solid curve) versus busulfan/treosulfan-based, *n* = 19 (dash curve). **b** Depending on the type of conditioning regimen: myeloablative conditioning (MAC) with irradiation, *n* = 10 (solid curve) versus reduced intensity conditioning (RIC) with irradiation, *n* = 12 (dash curve). **c** Depending on the presence of active infection at HSCT: with infection, *n* = 14 (dash curve) versus no infection, *n* = 34 (solid curve).

The OS after irradiation (*n* = 22) was 0.68 (95% CI: 0.48–0.88) and 0.63 (95% CI: 0.41–0.85) after busulfan/treosulfan-based (*n* = 19) conditioning, *p* = 0.66 (Fig. 2a). In the irradiation group, the OS was higher after MAC (*n* = 10) 0.9 (95% CI: 0.71–1.0) than RIC (*n* = 12) 0.49 (0.2–0.78), *p* = 0.05 (Fig. 2b). The OS was 0.68 (95% CI: 0.54–0.83) after TCRαβ/CD19 depleted HSCTs (*n* = 38) and 0.52 (95% CI: 0.17–0.86) after other HSCTs (*n* = 9), *p* = 0.58.

The OS in patients having active infection at second HSCT (*n* = 14) was 0.43 (95% CI: 0.17–0.69) versus 0.73 (95% CI: 0.58–0.88) in those without infection (*n* = 34), *p* = 0.004 (Fig. 2c). Absent autologous reconstitution after the first-transplant rejection (*n* = 11) did not affect post-second HSCT OS, which was 0.64 (95% CI: 0.35–0.92) versus 0.65 (95% CI: 0.49–0.80) in those with reconstituted hematopoiesis (*n* = 37), *p* = 0.76. The OS in the patients without symptoms of underlying disease (*n* = 32) was 0.63 (95% CI: 0.46–0.79) versus 0.69 (95% CI: 0.46–0.92) in those with disease symptoms (*n* = 16), *p* = 0.6. In patients without infections at second HSCT (*n* = 34), the OS was similar in patients having serious morbidity at HSCT (*n* = 12) 0.75 (95% CI: 0.51–1) and those without morbidity (*n* = 20) 0.73 (95% CI: 0.54–0.91), *p* = 0.96.

The patients with post-first HSCT uncontrolled AIHA and chronic skin GVHD are alive, asymptomatic and do not require immunosuppression. One patient with lung GVHD died early of infection and one patient with liver fibrosis due to GVHD, survived the HSCT, underwent liver transplantation, and died at day +406 of infection following poor immune recovery due to immunosuppression for liver allograft dysfunction.

### Last FU

Thirty one of 48 patients were alive at the time of analysis. From three patients alive after second HSCT GF, two received third HSCTs and at last FU were doing well and one with undefined HLH was waiting for the third HSCT with the disease controlled by ruxolitinib. Twenty seven of the other 28 (96%) patients at last FU did not have any disease symptoms. One patient with XIAP deficiency had mixed chimerism and inflammatory bowel disease controlled with adalimumab therapy.

Thirteen patients at last FU had various late HSCT complications: endocrine disorders in 4, growth delay in 4, kidney damage in 2, musculoskeletal system pathology in 5 (Table 3). Four patients after rituximab therapy for post-transplant complications require regular intravenous immunoglobulin therapy. Two patients had chronic lung GVHD and were on inhalation anti-inflammatory therapy, one of them had chronic skin GVHD on topical therapy.

**Table 3:** Late complications after conditioning regimens containing busulfan/treosulfan or irradiation.

Late complications	all	busulfan/treosulfan	irradiation
number of patients	9	6	3
median (range) FU time	4.8 (1.3–8.7)	5.12 (1.3–8.7)	2.1 (2–2.3)
median (range) age at FU	9.4 (5.6–15.4)	8.7 (6.2–15.4)	9.9 (5.6–11.7)
endocrine disorders	4	1	3
growth delay	4	4	–
kidney damage	2	2	–
musculoskeletal system pathology	5	5	–

*FU* follow up.

## Discussion

Second allogeneic HSCT remains the only salvage therapy for the patients developing GF. Despite the rising number of HSCT annually performed worldwide, there are no standard recommendations for the second transplant procedures. The common approach is to change conditioning regimen and use different donor, which, however, may be challenging. Very few studies report approaches and outcomes of second HSCT, especially in IEI.

The OS after the second HSCT in our group was 64%, while the second HSCT survival rate reported by the few small studies in non-malignant diseases varies from 77 to 90% [12–14]. The differences in OS between the studies may be explained by heterogeneous IEI included and patient selection for a second HSCT. In our center, re-transplantation as a salvage therapy has been given to all patients requiring this therapy, not considering the severity of co-morbidities, and 92% of those who developed GF after first HSCT received the second HSCT procedure. In our study, the only factor dramatically influencing the OS was uncontrolled infection at second HSCT. Interestingly, other complications such as absent autologous hematopoiesis reconstitution with high blood transfusion dependence and autoimmune complications of the IEI or second HSCT did not affect the second HSCT survival. Importantly, in two of our patients the second HSCT was effective and cured first transplant-related uncontrolled AIHA and chronic skin GVHD.

The choice of conditioning regimen for the second HSCT is challenging. In our center, conditioning regimen selection was based on the suspected mechanism of first transplant rejection, although in some cases it may not be clear. In suspected immune-mediated GF, for better immunoablation irradiation-based conditioning was chosen, and in suspected myeloid-mediated rejection, conditioning based on myeloablative doses busulfan or treosulfan was preferred. Radiotherapy is not commonly used in conditioning of IEI due to the additional risks of organ toxicity, particularly growth delay in younger patients [15], and the experience is limited to a few reports of low-dose total body irradiation [14, 16, 17]. 7 Gy lymphoid irradiation used pre-second HSCT in pediatric patients by the Tubingen group demonstrated sustained engraftment with quite a high rate of late complications [18]. Importantly, lower rates of late sequelae are expected after lower doses of irradiation [19, 20], and the doses used in our study may cause less toxicity. However, the incidence of late complications in our patients might be underestimated due to incomplete full organ assessment performed at last FU in some patients, young age of the patients to assess growth and gonadal function and short FU post-HSCT.

In our study, no difference in survival and late complications was seen between those who received irradiation- and busulfan/treosulfan-based conditioning. However, a higher mortality rate was observed after irradiation in combination with a RIC regimen, which can be explained by more severe pre-HSCT morbidity in the few patients and higher incidence of GF in this group. To potentiate the myeloablative effect of a busulfan/treosulfan, seven patients additionally received plerixafor [21], and none of them developed GF, although bigger groups are needed to study an effectiveness of such approach.

The common approach for second HSCT is to change the donor, however, there are no large studies in non-malignant diseases demonstrating differences in second HSCT outcomes depending the strategy of donor selection. Some small studies revealed no differences in HSCT results when the same donor was used [12]. In our center, the preferred strategy was to change the donor and 89% of the patients received a second transplant from a different donor. Certainly, a need of donor change has to be studied in larger groups of patients receiving the second transplant from both same and different donors.

TCRαβ/CD19 graft depletion in combination with pre-HSCT treosulfan demonstrated favorable outcomes in IEI with low risks of GVHD and transplant-related organ toxicity with either MUD or MMRD [9, 22]. Unfortunately, the increased risk of GF and a need of second HSCT procedures after TCRαβ/CD19 depletion remains a serious issue [9, 23]. At the same time, the low first transplant-related toxicity decreases the risk of second HSCT-associated toxicity, making the second HSCT more feasible. To reduce the risks of additional GVHD-related organ damage, TCRαβ/CD19 graft depletion was chosen in the majority of patients for a second HSCT procedure, and low rates of GVHD were seen. In our patients, the incidence of GF after the second HSCT was 0.15, although it seemed to be related to conditioning and was lower when MAC regimens were used.

One of a serious problems associated with TCRαβ/CD19 depleted grafts remain infectious complications [24]. In our study, most of the deaths were associated with infections, and the OS was significantly affected by pre-transplant infection. Novel strategies of infection prevention and treatment, sch as new anti-viral drugs or post-HSCT cell therapy may improve the results [25]. One of important doubts remains the timing of a second HSCT. Indeed, a longer period between two transplants might result in recovery from toxic effects of the first transplant and better outcome [13, 26, 27]. However, persistent BM aplasia and secondary immunodeficiency lead to increased risk of infections, therefore an individual decision based on the patient’s status must be made in every case. Another issue of infections associated with prolonged neutropenia may be the requirement of granulocyte transfusions, which can cause anti-HLA antibody production and increased risk of GF [28], which was observed in two of our patients.

The IEI are a big group of distinct disorders, affecting various mechanisms of immunity. Certainly, suspected risks of GF vary between different IEI and various levels of donor chimerism are needed to control different diseases [29–31]. There is a concern that the risk of GF is higher in patients with normal T-cell immunity than in combined IEI; however, in our group, patients with various IEI required the second HSCT and developed second transplant rejection. Larger groups of patients with specific molecular diagnoses are needed to estimate the risk of GF, although it seems this risk may be more related to the strategy of HSCT, rather than to underlying disorder [5, 8]. Interestingly, in our group, from the patients with suspected high transplant-related toxicity [7, 32] the patients with ataxia-telangiectasia, LIG4 deficiency and SDS did not survive, while two patients with Nijmegen breakage syndrome survived the second HSCT. Implementation of non-toxic myeloablative agents into conditioning regimens might decrease HSCT-related toxicity [10], which is of particular importance for IEI with DNA repair defects and SDS.

To conclude, we demonstrate generally favorable outcome for relatively large cohort of patients with IEI, who underwent second HSCT for GF. Larger studies in second HSCT are needed to propose recommendations for the second HSCT in IEI; although different underlying diseases, mechanisms of first graft rejection and patient status might not allow to completely unify the strategy. Longer FU and better patient assessment are needed to estimate the risks of late toxicity; however, selective use of TCRαβ/CD19 depleted grafts after conditioning regimens with low-dose lymphoid irradiation added to the myeloablative or sub-myeloablative regimes in patients with suspected immune-mediated first transplant GF may be a beneficial option for various IEI.

## Supplementary information

Table S1

## Data Availability

The datasets generated during and/or analysed during the current study are not publicly available due personal data protection policy, but are available from the corresponding author on reasonable request.
