# Supplementary material for: Second allogeneic hematopoietic stem cell transplantation in patients with inborn errors of immunity
Source: Bone Marrow Transplant. 2022 Dec 1;58(3):273–81. doi: 10.1038/s41409-022-01883-4 (PMC10005930; doi:10.1038/s41409-022-01883-4)
Supplement: Supplementary file 1 — Table S1 [file 41409_2022_1883_MOESM1_ESM.docx]

**Table S1. Characteristics of first HSCTs.**

| Patient № | Molecular diagnosis | Age at 1^st^ HSCT, years | Conditioning regimen | Donor (HLA-match) | Graft type | Post-HSCT immuno-suppression | Time of graft rejection, days post-HSCT | Type of graft rejection |
| --- | --- | --- | --- | --- | --- | --- | --- | --- |
| 1 | CD40L | 2.4 | Treo36, Flu150, ATGAM90 | MUD (10/10) | PBSC, TCRαβ depletion | Tacro, MMF | 160 | lymphoid |
| 2 | CYBB | 5.6 | Treo42, Flu150, ATGAM90 | MUD (10/10) | PBSC, TCRαβ depletion | Tacro | 140 | lymphoid |
| 3 | XIAP | 4.7 | Flu150, Mel140, Alemt1 | MRD (10/10) | BM | Tacro | 34 | - |
| 4 | ELANE | 9.0 | Treo42, Flu150, Thymo5 | MUD (10/10) | PBSC, TCRαβ depletion | Tacro, Mtx | PGF | - |
| 5 | CID undefined | 4.2 | Treo42, Flu150, ATGAM50 | MUD (9/10) | PBSC, TCRαβ depletion | Tacro, Mtx | 85 | lymphoid |
| 6 | STAT1 LOF | 6.3 | Treo42, Flu150, Thymo5, Rit | MUD (10/10) | PBSC, TCRαβ depletion | Tacro, Mtx, Abat | 34 | lymphoid |
| 7 | WAS | 2.0 | Treo42, Flu150, Thymo5, Rit | MUD (10/10) | PBSC, TCRαβ depletion | Tacro, Abat | 88 | - |
| 8 | IL2RG | 0.6 | Treo36, Flu150, ATGAM50 | MMRD (5/10) | PBSC, TCRαβ depletion | Tacro, Mtx | 301 | myeloid |
| 9 | ELANE | 1.1 | Treo36, Flu150, Thymo5, Mel140, Rit | MMRD (8/10) | PBSC, TCRαβ depletion | Tacro, Mtx | PGF | - |
| 10 | RMRP | 3.0 | Treo42, Flu150, Thymo5, Mel140, Rit | MUD (10/10) | PBSC, TCRαβ depletion | Tacro, Mtx | 31 | lymphoid |
| 11 | HLH undefined | 2.4 | Treo42, Flu150, Thymo5 | MMRD (6/10) | PBSC, TCRαβ depletion | Tacro | 33 | - |
| 12 | WAS | 0.9 | Treo36, Flu150, Cy100, Rit | MUD (9/10) | PBSC, TCRαβ depletion | Tacro, Mtx | 82 | myeloid |
| 13 | CYBB | 14.9 | Treo42, Flu150, Thymo5, Mel140, Rit | MUD (9/10) | PBSC, TCRαβ depletion | Tacro, Mtx | 63 | lymphoid |
| 14 | UNC13D | 1.2 | Treo36, Flu150, Thymo5, Rit | MUD (10/10) | PBSC, TCRαβ depletion | Tacro, Mtx | 83 | lymphoid |
| 15 | IL2RG | 2.7 | - | MMRD (5/10) | PBSC, TCRαβ depletion | Tacro | - | - |
| 16 | RAG1 | 0.3 | Treo36, Flu150, Alemt1 | MMRD (5/10) | PBSC, TCRαβ depletion | Tacro | 428 | - |
| 17 | CID undefined | 16.1 | Treo42, Flu150, Thymo5, Mel140, Rit | MUD (10/10) | PBSC, TCRαβ depletion | Tacro, Mtx | 152 | lymphoid |
| 18 | WAS | 1.1 | Treo42, Flu150, Mel140, Rit | MUD (10/10) | BM, Pt-Cy | Tacro | 153 | myeloid |
| 19 | WAS | 3.1 | Treo42, Flu150, Cy120, Thymo5, Rit | MMRD (6/10) | PBSC, TCRαβ depletion | Tacro | 52 | myeloid |
| 20 | WAS | 1.5 | Treo42, Flu150, Thymo5, Mel140, Rit | MUD (10/10) | PBSC, TCRαβ depletion | Tacro | 125 | myeloid |
| 21 | IL2RG | 1.0 | Treo36, Flu150, Thymo5, Rit | MMRD (5/10) | PBSC, TCRαβ depletion | Tacro | - | - |
| 22 | IL2RG | 1.0 | Treo36, Flu150, Rit | MUD (10/10) | PBSC, CD34 selection | Toc | 76 | myeloid |
| 23 | WAS | 2.0 | Treo42, Flu150, Thymo5, Mel140, Rit | MMRD (5/10) | PBSC, TCRαβ depletion | Tacro | 207 | myeloid |
| 24 | NBN | 4.9 | Bu4, Flu150, Cy40, Thymo5, Rit | MUD (10/10) | PBSC, TCRαβ depletion | Tacro | 181 | myeloid |
| 25 | NBN | 9.9 | Bu4, Flu150, Cy40, Thymo5, Rit | MUD (10/10) | PBSC, TCRαβ depletion | Tacro | 61 | lymphoid |
| 26 | IL2RG | 0,5 | Treo36, Thymo5, Plerix/G-CSF | MMRD (6/10) | PBSC, CD34 selection | Toc | 31 | - |
| 27 | CYBB | 2.8 | Treo42, Flu150, Mel140 | MUD (10/10) | BM, Pt-Cy | Tacro, MMF | 329 | myeloid |
| 28 | ELANE | 5.1 | Treo42, Flu150, Thymo5, Thio10, Rit | MUD (10/10) | PBSC, TCRαβ depletion | no | PGF | - |
| 29 | CTLA4 | 16.7 | Treo42, Flu150, Thymo5, Thio10, Rit | MUD (10/10) | PBSC, TCRαβ depletion | no | 56 | lymphoid |
| 30 | LIG4 | 8.7 | Flu100, Cy100, ATGAM100 | MRD (10/10) | BM | CsA, MMF, Abat | PGF | - |
| 31 | XIAP | 2.5 | Treo42, Flu150, Thymo5, Thio10, Rit | MMRD (7/10) | PBSC, TCRαβ depletion | no | 21 | - |
| 32 | KRAS | 3.4 | Treo42, Flu150, Thymo5, Thio10, Rit | MMRD (5/10) | PBSC, TCRαβ depletion | no | 17 | - |
| 33 | CXCR4 | 1.4 | Treo42, Flu150, Thymo5, Thio10, Rit | MUD (10/10) | PBSC, TCRαβ depletion | no | 27 | - |
| 34 | ATM | 1.0 | Treo20, Flu150, Cy40, Thymo5, Rit | MMRD (5/10) | PBSC, TCRαβ depletion | no | 19 | - |
| 35 | HLH undefined | 1.1 | Treo42, Flu150, Thymo5, Thio10, Rit | MMRD (7/10) | PBSC, TCRαβ depletion | no | 21 | - |
| 36 | IL2RG | 2.0 | Treo36, Thymo5, Plerix/G-CSF, Rit | MMRD (5/10) | PBSC, TCRαβ depletion | Abat, Toc* | 124 | myeloid |
| 37 | SCN undefined | 2.9 | Treo42, Flu150, Thymo5, Thio10, Rit | MUD (10/10) | PBSC, TCRαβ depletion | no | - | - |
| 38 | ELANE | 1.8 | Treo42, Flu150, Thymo5, Thio10, Rit | MMRD (5/10) | PBSC, TCRαβ depletion | no | PGF | lymphoid |
| 39 | CD40L | 1.3 | Treo42, Flu150, Thymo5, Mel140, Rit | MUD (10/10) | PBSC, TCRαβ depletion | no | 376 | lymphoid |
| 40 | PSTPIP1 | 9.2 | Treo42, Flu150, Thymo5, Mel140, Rit | MMRD (5/10) | PBSC, TCRαβ depletion | Toc | 17 | lymphoid |
| 41 | KRAS | 7.7 | Treo42, Flu150, Thymo5, Thio10, Rit | MMRD (5/10) | PBSC, TCRαβ depletion | no | PGF | - |
| 42 | SBDS | 16.9 | Treo42, Flu150, Thymo5, Thio10, Rit | MUD (10/10) | PBSC, TCRαβ depletion | Abat, Toc*, Bort | - | - |
| 43 | HLH undefined | 1.0 | Treo36, Flu150, Thymo5, Thio10, Rit | MMRD (5/10) | PBSC, TCRαβ depletion | Abat, Emap | PGF | - |
| 44 | WAS | 4.5 | Treo42, Flu150, Thymo5, Mel140, Plerix/G-CSF, Rit | MMRD (7/10) | PBSC, TCRαβ depletion | no | 201 | myeloid |
| 45 | GATA2 | 16.8 | Treo42, Flu150, Thymo5, Thio10, Rit | MMRD (7/10) | PBSC, TCRαβ depletion | no | PGF | - |
| 46 | WAS | 1.2 | Treo42, Flu150, Thymo5, Mel140, Plerix/G-CSF, Rit | MMRD (5/10) | PBSC, TCRαβ depletion | Tacro | - | - |
| 47 | GATA2 | 15.7 | Treo42, Flu150, Thymo5, Thio10, Rit | MMRD (5/10) | PBSC, TCRαβ depletion | no | 18 | lymphoid |
| 48 | SBDS | 15.0 | Treo42, Flu150, Thymo5, VP40, Rit | MMRD (6/10) | PBSC, TCRαβ depletion | no | PGF | - |

HLH – hemophagocytic lymphohistiocytosis, CID – combined immunodeficiency, SCN – severe congenital neutropenia, PGF – primary graft failure, BM – bone marrow, Cy – cyclophosphamide (mg/kg), Flu – fludarabine (mg/m^2^), Treo – treosulfan (g/m^2^), Bu – busulfan (mg/kg), VP – vepesid (mg/kg), Thymo – thymoglobulin (Genzyme Europe, mg/kg), Thio – thiotepa (mg/kg), Mel – melphalan (mg/m^2^), Plerix – plerixafor (0.72mg/kg), G-CSF – granulocyte colony-stimulating factor (50mg/kg), Alemt – Alemtuzumab (mg/kg), MUD – matched unrelated donor, MMRD – mismatched related donor, MRD – matched related donor, PBSC - peripheral blood stem cell, Pt-Cy – posttransplant cyclophosphamide (100mg/kg, day +3,4 post-HSCT), Rit – rituximab (100-375 mg/m^2^), Abat – abatacept (10 mg/kg days -1, +7, +14, +28), Tacro - tacrolimus, СsA - cyclosporin A, Мtx – methotrexate (10-15 mg/m2 days +1, +3, +6, +11); MMF – mycophenolate mofetil 30 mg/kg/day; Rux – ruxolitinib, Toc – tocilizumab (8mg/kg days -1, +14, +28, in P36 and 42 day -1), Bort – bortezomib (1.4 mg/m2 days -5, -2, +2,+5), Emap – emapalumab (10 mg/kg days -7, -1).
